# Preserving the endothelium in saphenous vein graft with both conventional and no-touch preparation

**DOI:** 10.1186/s13019-020-01352-3

**Published:** 2020-10-15

**Authors:** Toshiro Saito, Hiroshi Kurazumi, Ryo Suzuki, Yutaro Matsuno, Akihito Mikamo, Kimikazu Hamano

**Affiliations:** Department of Surgery and Clinical Science, Yamaguchi University Graduate School of Medicine, 1-1-1 Minami-Kogushi, Ube City, Yamaguchi 755-8505 Japan

**Keywords:** Coronary artery bypass grafting (CABG), Saphenous vein grafts (SVGs), Conventional preparation (CV), No-touch technique (NT), Endothelium

## Abstract

**Background:**

Despite the inferior patency compared to arterial grafts, a saphenous vein graft (SVG) is widely used for coronary artery bypass grafting (CABG). A lower atherosclerosis rate and higher patency have been reported for SVG obtained via the no-touch technique (NT) than via conventional preparation (CV). Although CV-mediated endothelial dysfunction is implied, the precise mechanism underlying the higher patency with NT is poorly understood.

**Methods:**

Human residual SVGs during CABG and SVG sections after autopsy were analyzed. The endothelial surface was observed using scanning electron microscopy (SEM) and blindly compared between CV and NT. The endothelial integrity was also analyzed with immunohistochemistry.

**Results:**

Unexpectedly, the hyperfine structure on SEM was comparable between CV and NT before grafting, and microvillus, a characteristic of endothelium, was indistinguishable between them. Von Willebrand Factor, an endothelial marker, was equally detected throughout the vascular wall in both groups from residual and postmortem sections.

**Conclusions:**

The morphological integrity of the endothelium was successfully preserved in SVG with CV, even at an ultrastructural level. Although its functionality remains to be addressed, other factors than the endothelium may be involved in the high patency obtained by NT. The present findings suggest that the characteristics of NT and surgical methodology should be reconsidered.

## Background

Coronary artery bypass grafting (CABG) is a standard therapy for ischemic heart disease. Despite the inferior patency compared to arterial grafts, saphenous vein grafts (SVGs) are still widely transplanted for CABG due to their ease of manipulation [1–4]. Consequently, the long-term patency of SVG has been thoroughly investigated.

Conventional preparation (CV) of SVG, consisting of deprivation of surrounding tissues and distension, has been utilized for decades but is implied to be associated with atherogenesis and a poor patency rate [5–8]. Since the 1990s, the no-touch technique (NT), which preserves the outer tissues without distension, has been recognized as the superior alternative to CV due to its salutary effect. Accumulating evidence indicates that SVGs harvested with NT exhibit lower rates of atherosclerosis and higher patency than those harvested via CV [9–12]. Many cardiac surgeons have an intense interest in the higher patency of NT-SVG and its mechanism. However, the detailed mechanisms underlying these effects remain unclear.

Based on the studies comparing CV and NT, it is now broadly accepted that conventional distension injures the endothelium and medial architecture, inducing inflammation and intimal proliferation [5–8]. In CV group, an injury to vasa vasorum may also downregulate the endothelial function. Partial denudation of the endothelium was reproducibly detected after CV [6, 8, 13, 14]. Distension-mediated endothelial damage and denudation may therefore be significantly involved in vein graft failure after CV. However, the distending pressure varies among surgeons, and an early study reported that only higher pressures (≥700 mmHg) cause endothelial damage [15]. Whether or not this concept predominantly accounts for the difference in atherosclerosis and graft patency rates between CV and NT therefore remains unclear. Furthermore, atherosclerosis may be caused by multiple factors, and factors such as the surrounding tissue composition or secreted molecules may play a crucial role in protecting NT-harvested SVGs against atherosclerosis.

To clarify the predominant mechanism by which NT provides its salutary effect, we investigated the morphological features of the endothelium in the residual SVG harvested by CV or NT during CABG through scanning electron microscopy (SEM). We commissioned the scans and analyses from unbiased observers. Subsequently, the surprising results reported by them encouraged us to conduct further analyses regarding the endothelial integrity of the SVGs.

## Methods

### Surgical aspects

All saphenous veins were examined by echography before surgery to determine their size, morphology and functionality. Veins with any abnormalies, such as regurgitation, varix or varicose conditions, were excluded from graft candidates. CABG was performed in an on-pump or off-pump fashion by the surgeon’s choice. There were no marked differences in the handling of SVGs between on- and off-pump procedures. In on-pump cases, the surgical procedure was performed under cardiopulmonary bypass with moderate hypothermia (28 to 30 °C) and cardiac arrest with tepid blood cardioplegia. Vein grafts were mainly bypassed to the right coronary artery (RCA) and left circumflex artery (LCX), and the left anterior descending artery was reconstructed by the internal mammary artery. Vein grafts were anastomosed to the RCA or LCX, followed by perfusion with tepid blood cardioplegia (on-pump) or blood (off-pump) and anastomosis of the other side to the aorta.

### SV harvesting techniques

1. CV group

The SV was exposed mainly from the lower leg by skipped longitudinal leg skin incisions, wherein the side branches were ligated. The vein was removed from the leg after dissection, connected to the cannula inserted into the femoral artery and dilated with arterial pressure for 10 min with blood mixed solution before the sewing of the anastomoses. The residual part of the vein was cut off from the central side (aortic side) after the anastomosis of the distal side to the RCA or LCX. In CV group, the vein was manually distended with blood mixed solution (30 ml blood, 3000 U of heparin sodium and 30 mg of papaverine hydrochloride) at < 300 mmHg. The adventitia was stripped off [16]. The harvested vein was stored in wet gauze at room temperature in an operating room. A few hours later, the stored vein was washed with PBS and fixed with specific buffer in our laboratory.

2) NT group

The SV was exposed via the same skin incisions as used for CV. The vein was isolated along with 5 mm of the surrounding fat tissue, and all visible side branches were ligated. After removal, the vein was connected to the cannula inserted into the femoral artery and dilated with arterial pressure for 10 min with blood mixed solution before the sewing of the anastomoses. The residual part of the vein was obtained as in the CV group. In the NT group, the vein was neither flushed nor distended manually. The adventitia was not removed. The harvested vein was stored and treated as in the CV group thereafter.

### *SVG pre-bypass samples* (from patient no. 1- no. 5)

The residual SVG samples were obtained from Patient No. 1 via CV, Patient No. 2 via NT, and Patient No. 3- No. 5 via both CV and NT. The endothelial surface of SVGs from CV group (No. 1 and No. 3) and NT group (No. 2 and No. 3) was analyzed with SEM. The endothelial integrity of SVGs from CV group (No. 1, No. 4 and No. 5) and NT group (No. 2, No.4 and No. 5) was analyzed with immunohistochemistry.

### *SVG autopsy samples* (from patient no. 6- no. 7)

1. Patient No. 6

The patient was a 75-year-old male. He had suffered acute myocardial infarction of the inferior wall. CAG revealed three-vessel disease, and he received CABG (on-pump, arrested heart) with LITA to LAD, SVGs to D1, 14PL, 4PD and 4PL. The CV technique was used to harvest SVGs. He ultimately died on day 8 after surgery due to low-output syndrome. The grafted SVG was harvested at the autopsy. Sample was prepared from the SVG to 14PL.

2) Patient No. 7

The patient was a 78-year-old male. He had suffered unstable angina. CAG revealed three-vessel disease, and he received off-pump CABG with RITA to LAD, LITA to 14PL, SVGs to D1, D2, 4PD and 4PL. The NT technique was used to harvest SVGs. He ultimately died on day 7 after surgery due to massive pulmonary embolism. The grafted SVG was harvested at the autopsy. Sample was prepared from the SVG to D1 and D2.

The patients’ characteristics are summarized below.

**Table:** 

Patient No.	Age	Sex	On−/Off- pump	Preparation	Analysis
1	71	M	On	CV	SEM and immunohistochemistry
2	73	F	Off	NT	SEM and immunohistochemistry
3	77	M	On	CV and NT	SEM
4	78	M	Off	CV and NT	immunohistochemistry
5	68	M	On	CV and NT	immunohistochemistry
6 (autopsy)	75	M	On	CV	immunohistochemistry
7 (autopsy)	78	M	Off	NT	immunohistochemistry

### SEM

The SEM analysis was conducted as described previously [17]. A few hours after resection, the SVG was fixed with 2% glutaraldehyde. We commissioned the scans and analyses from Hanaichi Ultrastructure Research Institute (Okazaki, Japan). The SVG section was washed, postfixed, dehydrated and examined by SEM (JSM-7500F; JEOL, Tokyo, Japan). We received the examination report from the company and also analyzed the images by ourselves.

### Immunohistochemistry

Immunohistochemistry was conducted as described previously [18]. A few hours after resection, the SVG was fixed with 10% formalin overnight at room temperature and embedded in paraffin. Tissue sections (3 μm) were stained with anti-von Willebrand Factor antibody (#65707; Cell Signaling Technology, Beverly, MA, U.S.A.). All images were captured by a BZ-X710 microscope (Keyence, Osaka, Japan) and analyzed using the Image J software program (National Institutes of Health).

## Results

**Fig. 1:**
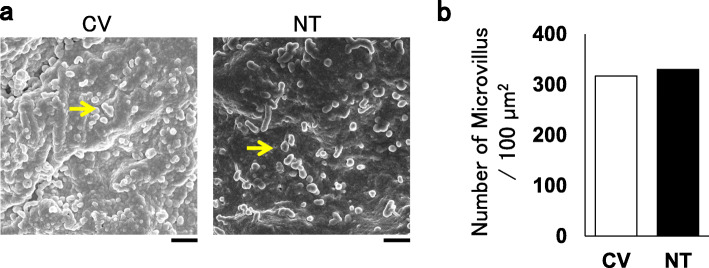
The inside of the residual SVG was observed with scanning microscopy. An unbiased observer reported no significant differences between the conventional (CV) and no-touch (NT) preparations. Representative images are shown in (**a**). The arrow indicates microvillus, a characteristic of endothelium. Scale bar, 1 μm. A summary is shown in (**b**). The average number of microvilli is indicated (*n* = 2).

The endothelial integrity of the residual SVG harvested by CV or NT before bypass-grafting was examined. Unexpectedly, the hyperfine structure on SEM was comparable between CV and NT before grafting (Fig. 1), and microvillus, a characteristic of endothelium, was observed in both cases, with a similar density and morphology (Fig. 1).

**Fig. 2:**
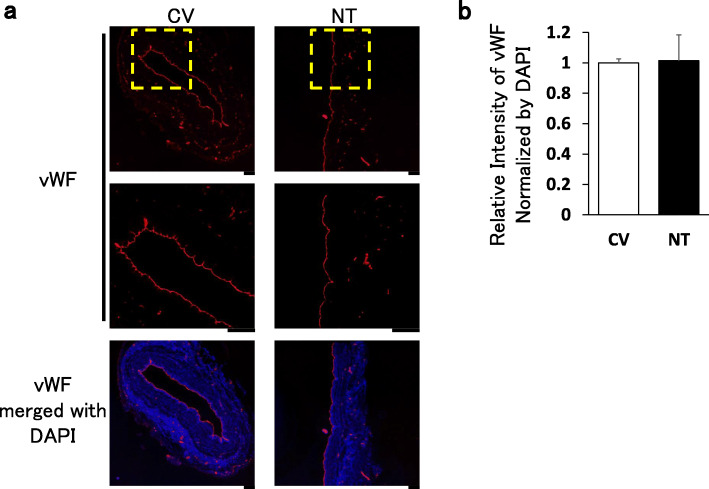
The endothelial integrity was assessed with immunohistochemistry in residual SVG. SVG was obtained with a conventional (CV) or no-touch (NT) approach during CABG. Representative stains of vWF (red) and DAPI (blue) are shown in (**a**). The demarcated area shows a higher magnification. Scale bar, 200 μm. A summary is shown in (**b**). The relative intensity of vWF normalized with that of DAPI is indicated (*n* = 3). Data are expressed as mean ± SEM.

Consistently, von Willebrand Factor (vWF), a representative marker of endothelial cell, was equally detected throughout the vascular wall in both groups (Fig. 2). There was no obvious defect or patch in the vWF signal throughout the entire luminal circumference of the CV group compared with the NT group. These results suggest that the morphological integrity of the endothelium was well maintained in the CV group even after pressure-mediated distension.

**Fig. 3:**
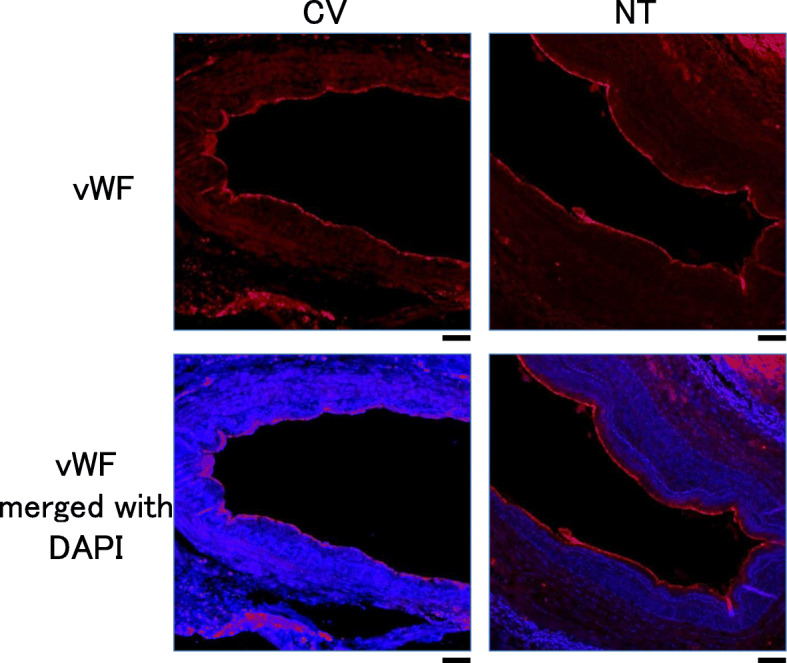
The endothelial integrity was assessed with immunohistochemistry in SVG sections obtained after an autopsy. SVG was obtained with a conventional (CV) or no-touch (NT) approach. Representative stains of vWF (red) and DAPI (blue) are shown. Scale bar, 100 μm.

Previous studies reported that high pressures induce endothelial inflammation after CV [5], suggesting that some of the endothelium may be lost in a later phase due to the accumulated damage. To address this issue, we collected autopsy sections of the SVG. The endothelium from 7 days after CV was compared with that from 8 days after NT. No marked differences were noted between them in the expression of vWF throughout the vascular wall (Fig. 3), suggesting that the endothelium was morphologically preserved after CV, even in the later phase.

## Discussion

In the present study, we clarified that the morphological integrity of the endothelium was successfully preserved in SVG with CV, even at an ultrastructural level. Although its functionality remains to be addressed, other factors than the endothelium may be involved in the high patency obtained by NT. The significance of the endothelium in mediating vascular homeostasis is generally well recognized, however, other factors are occasionally overlooked. Our study suggests that the characteristics of NT should be reconsidered, as this may innovatively stimulate the surgical methodology in the future.

One of the novel findings in this study is that the endothelial surface with microvillus was indistinguishable between CV and NT. To our knowledge, this is the first study comparing the hyperfine structure of the endothelial surface and microvillus between these procedures. Several previous studies used transmission electron microscopy (TEM) to observe SVG [19, 20]; our analysis using unbiased SEM is therefore unique.

Another novel point is our assessment of the endothelial integrity using postmortem sections. An early study with a large animal model noted that endothelial regeneration takes place as early as 7 days after denudation [16]. This finding thus suggests that even if endothelium is partially desquamated during CABG, the denuded lumina should become covered by newly generated endothelium within a short period of time. The endothelium we stained in the postmortem sections may have regenerated after denudation.

However, the present study possibly provides another hypothesis. Our data suggest that the endothelium after CV may be well preserved or only marginally damaged during CABG, showing a similar extent of damage to that after NT. Consequently, the remaining endothelium (rather than the regenerated tissue) may have been detected around the SVG wall in the postmortem sections.

There have been conflicting results regarding whether or not the endothelial integrity is preserved after pressure-mediated distension. In an earlier study involving the SEM analysis of the veins of monkey, distending pressure exceeding 700 mmHg induced morphological injury to the endothelium, while a lower pressure of 300–400 mmHg did not [15]. The endothelial morphology in veins subjected to this lower pressure was comparable to that in control veins lacking distension [15]. Conversely, studies with immunohistochemistry for CD31 using human SVGs demonstrated the partial loss of CD31 signaling after distension [6, 8]. Specifically, even after distension with a lower pressure of 100 mmHg, CD31 signaling was reduced by half, and the authors suggested that conventional distension significantly induced endothelial denudation [6].

While CD31 primarily localizes to the plasma membrane by exposing six extracellular domains on the cellular surface, vWF forms multimers in the endoplasmic reticulum and Golgi apparatus and is expressed in the cytosol before secretion [21–23]. As such, antigen of CD31 exposed on the endothelial surface may be vulnerable to distending pressure, thereby losing its reactivity to antibodies, whereas vWF in the cytosol may not be vulnerable. Although the hyperfine structure observed through SEM was comparable between the two groups in the present study, there might have been structural damages at a more hyperfine level on the endothelial surface in the CV group.

Since long-term patency is the ultimate goal of CABG, endothelial damage in the very acute phase may not be a critical for it. However, endothelial damage after distension is often discussed as if this is the predominant cause of poor patency in the CV group. The findings of the present study suggest the opposite due to the fact that there may be factors other than endothelium underlying the difference in the long-term patency between CV and NT.

### Limitations of the study

Since the immunoreactivity in the postmortem section is generally reduced during long-term storage, our ability to collect useful sections for analyses was limited. We therefore examined only a single postmortem section in each group.

## Conclusions

This study provides evidence that the endothelial hyperfine structure is well-preserved in SVGs harvested with CV. Other factors than the endothelium may play a pivotal role in protecting NT-harvested SVGs from atherosclerosis. Further investigations regarding the endothelial function and surrounding tissues will be required to elucidate the mechanism in detail.

## Data Availability

Not applicable.

## Abbreviations

CABG: Coronary artery bypass grafting
SVG: Saphenous vein graft
CV: Conventional preparation
NT: No-touch technique
SEM: Scanning electron microscopy
TEM: Transmission electron microscopy
